# The role of *Mycobacterium tuberculosis complex* species on apoptosis and necroptosis state of macrophages derived from active pulmonary tuberculosis patients

**DOI:** 10.1186/s13104-020-05256-2

**Published:** 2020-09-04

**Authors:** Budi Yanti, Mulyadi Mulyadi, Muhammad Amin, Harapan Harapan, Ni Made Mertaniasih, Soetjipto Soetjipto

**Affiliations:** 1 Postgraduate Program, Faculty of Medicine, Universitas Airlangga, Surabaya, Indonesia; 2 Department of Pulmonology and Respiratory Medicine, School of Medicine, Universitas Syiah Kuala, Jl. T. Tanoeh Abe, Darussalam, Banda Aceh, 23111 Indonesia; 3 Department of Internal Medicine, Faculty of Medicine, Universitas Nahdhatul Ulama Surabaya, Surabaya, Indonesia; 4 Department of Pulmonology and Respiratory Medicine, Faculty of Medicine, Universitas Airlangga, Surabaya, Indonesia; 5 Medical Research Unit, School of Medicine, Universitas Syiah Kuala, Banda Aceh, Indonesia; 6 Department of Microbiology, School of Medicine, Universitas Syiah Kuala, Banda Aceh, Indonesia; 7 Department of Clinical Microbiology, Faculty of Medicine, Universitas Airlangga, Surabaya, Indonesia; 8 Institute of Tropical Diseases, Universitas Airlangga, Surabaya, Indonesia; 9 Department of Medical Biochemistry, Faculty of Medicine, Universitas Airlangga, Kampus C Mulyorejo Kec. Mulyorejo–Kota, Surabaya, Prov. Jawa Timur 60115 Indonesia

**Keywords:** *Mycobacterium tuberculosis*, *Mycobacterium bovis*, Apoptosis, Necroptosis, FADD, RIP3

## Abstract

**Objective:**

The role of *Mycobacterium tuberculosis* complex (MTBC) species in tuberculosis (TB) infection in human is still questioned. The aim of this study was to determine whether *M. tuberculosis* and *M. bovis* is associated with apoptosis and necroptosis by measuring the expression of specific signaling pathways components (Fas-associated protein with death domain (FADD) and receptor interacting protein 3 (RIP3)), and the level of apoptosis.

**Results:**

We recruited 30 patients with pulmonary TB; 24 patients were infected with *M. tuberculosis* Beijing strain and six patients with *M. bovis* BCG strain. *M. tuberculosis*-infected patients were more likely to have severe lung damage compared to those infected with *M. bovis (*odds ratio [OR] 7.60; 95% confidence interval [CI] 1.07–54.09). *M. tuberculosis* infection was associated with lower expression of FADD and lower apoptosis level of macrophages compared to *M. bovis*. No significant different of RIP3 between MTBC species groups. In conclusion, *M. tuberculosis* Beijing strain was associated with severe pulmonary damage, inhibited FADD expression and reduced apoptosis level of macrophages derived from pulmonary TB patients. This suggests that the *M. tuberculosis* Beijing strain is potentially to be used as determinant of disease progressivity and tissue damage in TB cases.

## Introduction

*Mycobacterium tuberculosis* complex *(*MTBC) continues to significantly impact public health and is associated with one million deaths of tuberculosis (TB) cases annually worldwide [1]. Ability of *M. tuberculosis* to establish disease is entirely depend on macrophage deaths during infection. Pulmonary macrophages are critical component of the primary innate immune response that have various functions in immune surveillances, removal of cellular debris, microbial clearance, and in resolution of inflammation [2]. There are two pathways of macrophage deaths, apoptosis and necroptosis, that are developed as host antimicrobial defenses in the early TB infection; both of them are programmed cell death [3]. These mechanisms are triggered by tumor necrosis factor alpha (TNFα), oxidative stress, lipopolysaccharide (LPS), and other factors [4]. Apoptosis is characterized by signaling cell through Fas-associated protein with death domain (FADD), a crucial protein that is associated with death receptors (DRs) [5]. Necroptosis can be induced if apoptotic signaling is inhibited through formation of receptor interacting protein 3 (RIP3) [6, 7].

MTBC comprises of many members including *M. tuberculosis*, *M. africanum, M. canettii, M. bovis, M. microti, M. orygis, M. caprae, M. pinnipedii, M. suricattae* and *M. mungi)* [8]. These members have different cellular components, the ability of human-to-human transmission, and severity of disease [9]. *M. bovis* lacks of trehalose-containing glycolipids on its cell walls that could affect the virulence and adaptability within the host cells. The genetic analysis showed that the loss of trehalose-containing glycolipids was related to disturbance surface-exposed acyltrehaloses such sulfatides (SLs), diacyltrehaloses (DATs), triacyltrehaloses (TATs) and pentacyltrehaloses (PATs) and the phoPR component signaling system [10, 11]. *In M. tuberculosis*, this PhoPR system plays a role in the regulation of cell wall complex lipid biosynthesis and the secretion of EsxA/ESAT-6 for modulating the immune response [12]. Reduced this signaling system in *M. bovis* has been linked to less virulence in humans [11]. Another study showed that MTBC species with dominant PhoP gene expression are hypervirulent and resistant to tuberculosis drugs [13].The role of MTBC species have been proven in various animal models [14], but still be questioned in human [9]. Although some species have 99.9% similarity of nucleotide sequences, they have different abilities to induce macrophages death [15]. Apoptosis and necroptosis play the important roles in innate immune responses against pathogens [16] and are crucial in TB infection [17, 18]. In vitro studies showed that the apoptosis of BCG-infected monocytes by the exogenous drug was associated with a reduction of bacillary viability while necrosis was not associated with reduction of BCG viability [19, 20]. Another study found that if apoptosis was predominated during a TB infection the bacteria were potentially to be cleared [21]. *M tuberculosis* Beijing strain with high virulent inhibits apoptosis, and triggers necroptosis because it evades the immune system, induces the necrosis, lyses of the cellular components, and induces the parenchymal destruction and therefore is associated with severe TB [22]. The aim of this study was to assess the role of *M. tuberculosis* and *M. bovis* on the state of apoptotic and necroptosis of macrophages isolated from TB patients.

## Main text

### Method

#### Study setting and patients

Between June and October 2017, a cross-sectional study was conducted. Confirmed new pulmonary TB cases were recruited from Tuberculosis Clinic at Soewandhie Hospital, Surabaya, Indonesia. Bacteriological confirmation was conducted by sputum acid fast staining and GeneXpert MTB/RIF test (Cepheid, Sunnyvale, CA, USA). For the study purpose, the patients underwent fiber optic bronchoscopy to collect bronchoalveolar lavage fluid (BALF) and the macrophages were collected from the BALF. Patients with HIV co-infection, diabetes mellitus, renal abnormality, heart diseases, immune response disorders such as lupus erythematosus and rheumatoid arthritis, non-TB pulmonary diseases, and those who previously received anti-TB treatment were excluded. All samples were tested to identify MTBC species using polymerase chain reaction (PCR) targeting two specific genes: RD9 and TbD1.

#### Assessment of pulmonary damage

The degree of pulmonary damage was classified using the NICE Scoring System based on the total lesions in six lung areas [23]. This system assessed four components: the nodule (N), the infiltration or consolidation (I), the cavity (C), and the ectasis (E) based on chest radiograph of three areas of each lung (i.e. six areas of both lungs). For each area, the possible scores were 1 to 4 indicating the lung damage area of 0–25%, > 25%– ≤ 50%, > 50%– ≤ 75% and > 75%, respectively. The pulmonary damage was then categorized as mild if the total score was 8 or less and severe if the total score was more than 8.

#### Samples collection and macrophages isolation

BAL was performed using 10 ml of saline solution as described previously [24]. The BALF was centrifuged at 2500 rpm for 15 min, the supernatant was discarded, and cells were resuspended to a cell count of 4 × 10^5^ cells/ml with RPMI 1640 medium. The total cell count was measured using hemocytometer.

#### FADD and RIP3 expression by immunocytochemical staining

Pellet cells derived from the centrifugation were applied to glass slides and then washed with PBS three times for 10 min. Permeabilization was performed with a CA-630-0.5% Igepal solution (Sigma Aldrich, Saint Louis, MO, USA). H_2_O_2_ 0.3% was then added and incubated for 10 min before was washed with PBS. The slides were incubated with anti-human monoclonal antibody FADD or RIP3 followed manufacturer’s protocol (Santa Cruz, Oregon, OR, USA). The quantification of the protein expression was conducted according to the previous study [25].

#### Apoptosis assay

The level of apoptosis in infected macrophages was determined by using the Tunel Assay apoptosis kit per manufacturer’s protocol (R&D Systems, Minneapolis, MN, USA). Tunel assay was performed with terminal deoxynucleotidyl transferase enzymes to determine the fragmentation of DNA. The level of apoptosis was measured based on the previous study [26].

#### MTBC Species identification and sequence confirmation

The detection of MTBC species was conducted from the BALF. Briefly, DNA was extracted using DNeasy^®^ Blood & Tissue kit (Ambion Inc., Austin, TX, USA). Amplification of gene-specific *M. tuberculosis* was conducted using RD9 primers (F: 5′-GTGTAGGTCAGCCCCATCC-3′, I: 5-CAATGTTTGTTGCGCTGC-3′, R: 5′-GCTACCCTCGACCAAGTGTT-3′), while *M. bovis* was identified using TbD1 primers (F: 5′-AGTGACTGGCCTGGTCAAAC-3′, R: 5′-GAGCTCTGTGCGACGTTATG-3′) [27, 28]. The conditions for PCR assays were set up for 30 s at 94 °C (denaturation), followed by 35 cycles of denaturation (94 °C, 30 s), annealing (56 °C, 1 s), and extension (72 °C, 10 min). The confirmation of the strain was conducted by sequencing nine and two of *M. tuberculosis* and *M. bovis* samples, respectively and the homology analysis was conducted using Basic Local Alignment Search Tool (BLAST).

#### Statistical analysis

Associations between MTBC species and the degree of lung damage including for each subset of NICE component were assessed using Chi squared test. To compare the level of apoptosis, FADD, and RIP3 of macrophages between *M. tuberculosis* and *M. bovis* groups, the Man-Whitney test was employed. For all analyses, significance was assessed at α = 0.05.

### Results

#### Characteristics of patients

Forty new active pulmonary TB patients were successfully diagnosed and met the inclusion criteria and 30 patients were willing to participate and underwent the BAL procedure. Among 30 patients, majority of them (81.37%) were female and more than half (16/30, 53.3%) aged between 21 and 40 years old (Table 1). Majority of the patients (75%) were working as laborer and five patients (16.6%) were working as cow slaughters. Based on clinical symptoms, 90%, 86%, 56% and of the patients had anorexia, experienced weight loss, and had persistent fever, respectively. Only 36.6% of patients had low hemoglobin level and 30.0% had low oxygen saturation.

**Table 1:** Demographic and clinical characteristics between *M. tuberculosis* Beijing strain and *M. bovis* BCG strain

Variable	MTBC species		*p* value
	*M. tuberculosis* Beijing strain, n (%)	*M. bovis* BCG strain, n (%)	
Gender			
Female	13 (81.37)	3 (18.8)	0.855
Male	11 (78.6)	3 (21.4)	
Age (year)			
< 21	2 (50.0)	2 (50.0)	0.172
21–40	12 (75.0)	4 (25.0)	
40–50	6 (100.0)	0 (0)	
> 50	4 (100.0)	0 (0)	
Educational attainment			
Elementary school	8 (89.5)	1 (11.1)	0.466
Junior high school	10 (83.3)	2 (6.7)	
Senior high school	6 (66.7)	3 (33.3)	
Occupation			
Labourer	16 (76.2)	5 (32.8)	0.364
Housewife	6 (100.0)	0 (0)	
Unemployed	2 (66.7)	1 (33.3)	
Anorexia			
Yes	21 (77.7)	6 (22.2)	0.189
No	3 (100.0)	0 (0)	
Weight loss			
Yes	21 (80.8)	5 (19.2)	0.364
No	3 (75.0)	1 (25.0)	
Fever			
Yes	13 (76.5)	4 (23.5)	0.167
No	11 (84.6)	2 (15.4)	
Haemoglobin level			
Normal	8 (72.8)	3 (27.3)	0.750
Low	16 (84.2)	3 (15.8)	
SaO_2_ level			
Normal	17 (80.9)	4 (19.0)	0.831
Low	7 (77.8)	2 (22.2)	

#### Detection of MTBC species

Based on RD9 gene amplification, 24 (80.0%) *M. tuberculosis* were identified and nine of them were sequenced for the confirmation. The isolates had 99–100% sequence similarity with the *M. tuberculosis* Beijing strain 2014 PNGD (Accession no CP022704.2). Six (20.0%) *M. bovis* were identified and two isolates were sequenced. All of them had 100% sequence similarity with *M. bovis* BCG strain (Accession no CP033311.1).

#### Association between MTBC species and lung damage

MTBC species had no association with three NICE components (i.e. the presence of nodule, the infiltrate or consolidation, and the cavity of the lungs) (Table 2). Ectasis, however, was more frequent in *M. tuberculosis* (OR: 10.0; 95% CI 1.34–74.51). *M. tuberculosis* was identified in 19 (90.50%) patients with severe lung damage. There was a significant association between *M. tuberculosis* and severe lung tissue damage, OR: 7.60; 95% CI 1.07–54.09, p = 0.028 (Table 2).

**Table 2:** Severity of pulmonary damage between *M. tuberculosis* Beijing strain and *M. bovis* BCG strain

Variables	*n*	MTBC species		OR	95% CI	p-value
		*M. tuberculosis* Beijing strain, n (%)	*M. bovis* BCG strain, n (%)			
NICE score						
Nodule				4.85	0.72–32.87	0.088
Yes	19	17 (89.5)	2 (10.5)			
No	11	7 (63.7)	4 (36.4)			
Infiltrate/consolidation				NA	NA	NA
Yes	30	24 (80.0)	6 (20.0)			
No	0	0 (0.0)	0 (0.0)			
Cavitas				NA	NA	0.283
Yes	4	4 (100.0)	0 (0.0)			
No	26	20 (76.9)	6 (23.1)			
Ectasis				10.00	1.34–74.51	0.013
Yes	22	20 (90.0)	2 (9.15)			
No	8	4 (50.0)	4 (50.0)			
Severity of lung damage				7.60	1.07–54.09	0.028
Mild	9	5 (9.5)	4 (55.6)			
Severe	21	19 (90.5)	2 (44.4)			

#### Association between MTBC species and FADD, RIP3, and apoptosis

**Fig. 1:**
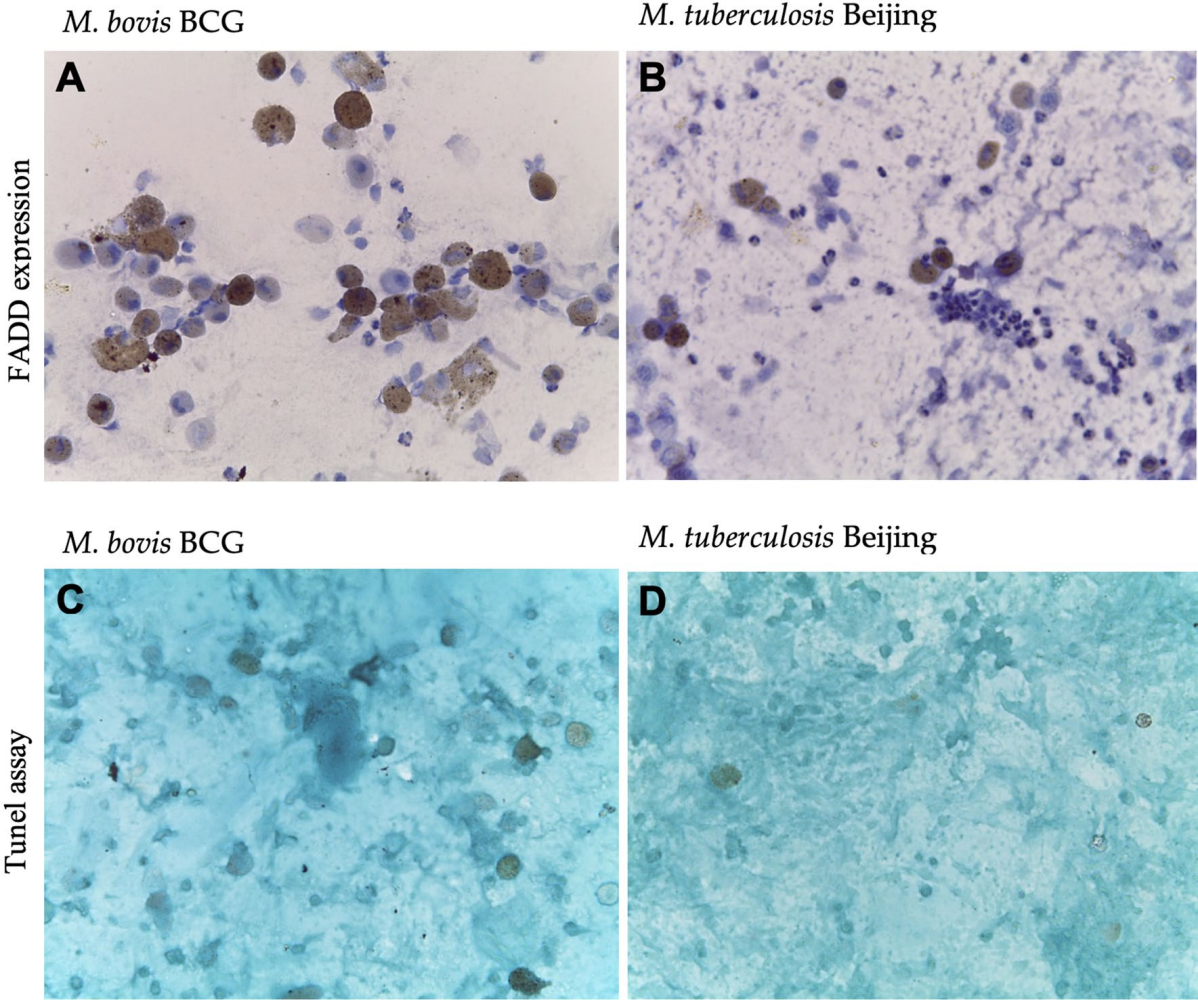
FADD expression (**a**, **b**) and apoptosis (**c**, **d**) of macrophages derived from active pulmonary tuberculosis patients infected with *M. bovis* BCG strain (**a**, **c**) and *M. tuberculosis* Beijing strain (**b**, **d**)

Our data indicated that the level of FADD was lower in *M tuberculosis* group compared to *M. bovis*, 0.208 ± 1.020 vs. 0.667 ± 1.032 cells with *p *= 0.046 (see Fig. 1a, b). The level of RIP3 expression was not different between *M tuberculosis* group and *M. bovis (*0.333 ± 0.702 vs 0.500 ± 0.836, p = 0.551). Data from Tunel assay indicated that the level of apoptosis in macrophages derived from *M tuberculosis* group was significantly lower compared to *M. bovis* group, 0.875 ± 1.676 vs. 2.500 ± 3.331, *p *= 0.049 (Fig. 1c, d).

### Discussion

The outcome and the disease progression of MTBC species infection are varied; exposure to this mycobacterium can be rapidly cleared by innate immunity or direct progression to active TB. Active TB also has a range of presentations and each form is associated with diverse host responses to the pathogen. Studies have provided evidence that different MTBC species is associated with different virulent [29–31] and would affect host–pathogen interactions [32]. Phenotypic comparisons between *M. tuberculosis* and *M. bovis* have been limited to animal studies, which suggested that *M. bovis* is likely less virulent [9, 33, 34]. In the present study, 80.0% of TB cases caused by *M. tuberculosis* and inhibited the cell signaling to apoptosis execution. The previous studies have reported that high virulent *M. tuberculosis* inhibited apoptosis in TB-cases [35, 36]. Virulent *M. tuberculosis* H37Rv and Erdman for example inhibited apoptosis stronger compared to non-virulent *M. bovis* BCG strain, H37Ra, and *M. kansaii* on human alveolar macrophages of healthy nonsmoking volunteers [36]. Other studies found that *M. tuberculosis* inhibited and suppressed apoptosis of host macrophages on THP-1 [37, 38] and J774 cell lines [39].

Data from the present study identified that infection of macrophages with *M. tuberculosis* was associated with a lower level of FADD compared to *M. bovis* infection. FADD is an adapter protein to bind caspase 8 and caspase 10 precursors and is simultaneously activated and mediated cell signals with caspases 3, 6, and 7 to induce apoptosis [40]. This suggests that *M. tuberculosis* is able to inhibit signaling of caspases to execute the apoptosis. A study showed that low FADD expression triggered the necrosis [41] and the necroptosis [42]. Altogether, these explain, in part, the finding of present study that *M. tuberculosis* infection was significantly associated with severe lung damage.

In conclusion, our preeliminary data suggest that *M. tuberculosis* is associated with more severe lung damage compared to *M. bovis* infection. *M. tuberculosis* also inhibits FADD expression and reduces the apoptosis level.

#### Study limitation

This was a cross-sectional study at a single health center and included small number of pulmonary TB patients determined to be infected predominantly with *M. tuberculosis* Beijing strains. Therefore, our study was underpowered, which lessened its internal validity. In this study, the FADD expression was used which may not be the best marker for propensity towards apoptosis or necrosis. Therefore, validation using other standard approaches such as caspase-activity and RIP3 phosphorylation is warrant. Finally, we did not assess the necrosis state of the cells and further study to analysis the role of MTBC species on necrosis is therefore also important.

## Data Availability

The datasets generated during and/or analyzed during the current study are available from the corresponding author on reasonable request.

## Abbreviations

BALF: Bronchoalveolar lavage fluid
CI: Confidence interval
DRs: Death receptors
FADD: Fas-associated protein with death domain
LPS: Lipopolysaccharide
MTBC: *Mycobacterium tuberculosis* complex
OR: Odds ratio
PCR: Polymerase chain reaction
RIP3: Receptor interacting protein 3
TB: Tuberculosis
TNFα: Tumor necrosis factor alpha
